# Longitudinal Study after Sputnik V Vaccination Shows Durable SARS-CoV-2 Neutralizing Antibodies and Reduced Viral Variant Escape to Neutralization over Time

**DOI:** 10.1128/mbio.03442-21

**Published:** 2022-01-25

**Authors:** María M. Gonzalez Lopez Ledesma, Lautaro Sanchez, Diego S. Ojeda, Santiago Oviedo Rouco, Andrés H. Rossi, Augusto Varese, Ignacio Mazzitelli, Carla A. Pascuale, Esteban A. Miglietta, Pamela E. Rodríguez, Horacio M. Pallarés, Guadalupe S. Costa Navarro, Julio J. Caramelo, Paul W. Rothlauf, Zhuoming Liu, Louis-Marie Bloyet, Marjorie Cornejo Pontelli, Natali B. Rasetto, Shirley D. Wenker, Lila Y. Ramis, Magalí G. Bialer, María Jose de Leone, C. Esteban Hernando, Luciana Bianchimano, Antonella S. Ríos, María Soledad Treffinger Cienfuegos, Diana R. Rodriguez García, Yesica Longueira, Natalia Laufer, Diego Alvarez, Ana Ceballos, Valeria Ochoa, Cecilia Monzani, Gariela Turk, Melina Salvatori, Jorge Carradori, Katherine Prost, Alejandra Rima, Claudia Varela, Regina Ercole, Rosana I. Toro, Sebastian Gutierrez, Martín Zubieta, Dolores Acuña, Mercedes S. Nabaes Jodar, Carolina Torres, Laura Mojsiejczuk, Mariana Viegas, Pilar Velazquez, Clarisa Testa, Nicolas Kreplak, Marcelo Yanovsky, Sean Whelan, Jorge Geffner, Marina Pifano, Andrea V. Gamarnik

**Affiliations:** a Fundación Instituto Leloir-CONICET, Buenos Aires, Argentina; b Universidad de Buenos Aires, Facultad de Medicina, Instituto de Investigaciones Biomédicas en Retrovirus y SIDA (INBIRS-CONICET), Buenos Aires, Argentina; c Department of Molecular Microbiology, Washington University School of Medicine, St. Louis, Missouri, USA; d Biobanco de Enfermedades Infecciosas (INBIRS-UBA-CONICET), Buenos Aires, Argentina; e Instituto de Investigaciones Biotecnológicas, UNSAM-CONICET, Buenos Aires, Argentina; f Laboratorio Lemos S.R.L., Buenos Aires, Argentina; g Hospital Interzonal General de Agudos Dr Pedro Fiorito, Buenos Aires, Argentina; h Hospital Interzonal General de Agudos Evita, Buenos Aires, Argentina; i Hospital Interzonal General de Agudos Prof. Dr. Rodolfo Rossi, Buenos Aires, Argentina; j Hospital Interzonal Especializado de Agudos y Crónicos San Juan de Dios, Buenos Aires, Argentina; k Hospital Interzonal General de Agudos San Roque, Buenos Aires, Argentina; l Hospital Interzonal General de Agudos San Martín, Buenos Aires, Argentina; m Hospital de Alta Complejidad El Cruce “Nestor Kirchner,” Buenos Aires, Argentina; n Hospital General de Niños Dr. Ricardo Gutierrez e Instituto de Investigaciones en Bacteriología y Virología Molecular, Fac de Farmacia y Bioquímica, UBA, Buenos Aires, Argentina; o Ministerio de Salud de Provincia de Buenos Aires, Buenos Aires, Argentina; Duke University School of Medicine

**Keywords:** COVID-19, SARS CoV-2, Sputnik V, viral variants

## Abstract

Recent studies have shown a temporal increase in the neutralizing antibody potency and breadth to SARS-CoV-2 variants in coronavirus disease 2019 (COVID-19) convalescent individuals. Here, we examined longitudinal antibody responses and viral neutralizing capacity to the B.1 lineage virus (Wuhan related), to variants of concern (VOC; Alpha, Beta, Gamma, and Delta), and to a local variant of interest (VOI; Lambda) in volunteers receiving the Sputnik V vaccine in Argentina. Longitudinal serum samples (*N* = 536) collected from 118 volunteers obtained between January and October 2021 were used. The analysis indicates that while anti-spike IgG levels significantly wane over time, the neutralizing capacity for the Wuhan-related lineages of SARS-CoV-2 and VOC is maintained within 6 months of vaccination. In addition, an improved antibody cross-neutralizing ability for circulating variants of concern (Beta and Gamma) was observed over time postvaccination. The viral variants that displayed higher escape to neutralizing antibodies with respect to the original virus (Beta and Gamma variants) were the ones showing the largest increase in susceptibility to neutralization over time after vaccination. Our observations indicate that serum neutralizing antibodies are maintained for at least 6 months and show a reduction of VOC escape to neutralizing antibodies over time after vaccination.

## INTRODUCTION

The coronavirus disease 2019 (COVID-19) pandemic is devastating economies and health care systems worldwide and had caused more than 5 million deaths by November 2021 (1). Mass vaccination offers the possibility of halting this global burden. However, the limited vaccine supply and inequalities in vaccine accessibility create a need to increase international cooperation. An additional challenge in combatting COVID-19 has been the emergence in late 2020 of new viral variants around the world with greater transmissibility, replication, and/or resistance to neutralizing antibodies. Viral variants harboring mutations in the spike protein may compromise vaccine immune control, and the rapid spread of these variants could undermine current efforts to end the pandemic. Thus, constant surveillance of viral variant emergence and documentation of immune responses elicited by different vaccine platforms are fundamental to optimizing pandemic control measures.

The humoral immune response elicited by SARS-CoV-2 infection and vaccination is a relevant marker of protection against subsequent viral encounters (2–4). Recent reports have provided important information regarding antibody durability and maturation processes in infected patients (5–7). Antibody titers against SARS-CoV-2 were shown to wane over time for COVID-19 convalescent individuals, while antibody maturation increased the neutralization potency to the original SARS-CoV-2 and variants of concern (VOC) (8, 9). This phenomenon has not been well described for vaccinated individuals.

In this study, we evaluated the humoral response over time and the neutralizing potency of antibodies elicited by Sputnik V vaccination in Argentina. Sputnik V (Gam-COVID-Vac) has been extensively used in Argentina and in more than 65 countries around the world. It consists of a two-component heterologous recombinant adenovirus-based vaccine (rAd type 26 and rAd type 5) expressing the spike protein (10, 11). Evaluation of the immune response over time up to 6 months after vaccination indicates that anti-spike antibody levels wane but neutralization capacity is maintained not only for the ancestral SARS-CoV-2 but also for widely and locally circulating viral variants. These data support the notion that antibody affinity maturation and limitation of VOC escape to neutralization occur over time after Sputnik V vaccination.

## RESULTS

We evaluated the longitudinal anti-spike IgG antibody level and viral neutralizing capacity to SARS-CoV-2 VOC in 118 volunteers (see Table S1 in the supplemental material) receiving the complete two-dose regimen of the Sputnik V vaccine in Argentina as a continuation of our recent report (12). A collection of 536 serum samples were initially obtained between January and October 2021. Plasma samples were taken at five time points: before vaccination (baseline) and at 21, 42, 120, and 180 days after the initial vaccination (second dose was applied at 21 days). The levels of IgG antibodies against the complete original Wuhan spike protein were measured by titration (13), and quantification was carried out using the WHO International Antibody Standard (14). According to the presence of antibodies at baseline, samples were divided in two groups, without (group 1) or with (group 2) previous SARS-CoV-2 infection. Evaluation of infection during the study was assessed by measuring the presence of IgG antinucleocapsid at 42, 120, and 180 days after Sputnik V vaccination. This study indicated that 3 individuals were infected during that time. Thus, they were removed from the analysis. Virus-neutralizing antibodies were evaluated using two systems, a pseudotyped vesicular stomatitis virus (VSV) expressing green fluorescent protein (GFP) (15), carrying spike from the Wuhan, Alpha (United Kingdom), Beta (South Africa), Gamma (Manaos), and Delta (India) variants, and the SARS-CoV-2 virus using local isolates for the B.1 lineage (Wuhan related) and regional circulating SARS-CoV-2 variants (Alpha, Gamma, and Lambda).

10.1128/mbio.03442-21.2 TABLE S1 Patient information: age, gender, and group classification. Download Table S1, DOCX file, 0.02 MB. Copyright © 2022 Gonzalez Lopez Ledesma et al. 2022 Gonzalez Lopez Ledesma et al. https://creativecommons.org/licenses/by/4.0/ This content is distributed under the terms of the Creative Commons Attribution 4.0 International license.

### Sustained viral neutralization capacity over time upon Sputnik V vaccination.

**FIG 1:**
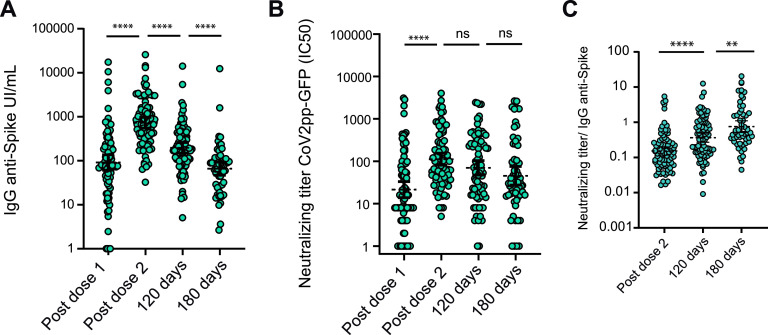
Immune response to the Sputnik V vaccine in naive participants. (A) IgG anti-spike antibody levels quantified according to the WHO International Antibody Standard (*N *=* *88). Antibodies were measured 21 (*N *=* *88), 42 (21 days after second dose) (*N *=* *88), 120 (*N *=* *88), and 180 (*N* = 64) days after the initial vaccination. (B) Neutralizing titers measured at 50% inhibition against the pseudotyped virus (CoV2pp GFP) for the same cohort as in panel A. The geometric means with 95% confidence intervals are shown. Wilcoxon matched-pair test was used. Statistical significance is shown with the following notations: ****, *P < *0.0001; ***, *P < *0.001; ns, not significant. (C) Neutralizing index calculated at 42, 120, and 180 days after the initial vaccination. The index was defined as the neutralizing titer/IgG anti-spike measurement for each participant.

A longitudinal analysis (*N *= 536) from 118 volunteers vaccinated with the two-dose regimen of Sputnik V showed that IgG levels declined over a period of 6 months, but all the samples analyzed remained seropositive. The geometric mean (GM) international units of IgG anti-spike antibodies per milliliter (IU/ml) for the group that was seronegative (naive) at baseline (group 1, *N *=* *88) declined from 732 (95% confidence intervals [95% CI], 552 to 959) at 42 days to 196.9 (95% CI, 149 to 260) and 64 (95% CI, 46 to 90) by 120 and 180 days, respectively, after the initial vaccination (Fig. 1A). IgG level waning was also observed in participants who were seropositive (due to prior infection) at baseline (group 2; Fig. S1). For this group, the GM of antibody (IU/ml) was the highest after the first dose of the vaccine, 9,429 (95% CI, 6,303 to 14,105), and declined to 5,193 (95% CI, 3,390 to 7,960) and 2,719 (95% CI, 1,706 to 4,333) at 42 and 120 days after the initial vaccination. The geometric mean half-maximal neutralizing titer (GMT IC_50_) of group 1 samples at 42, 120, and 180 days after the initial vaccination was 112 (95% CI, 80 to 155), 70 (95% CI, 47 to 103), and 44 (95% CI, 26 to 73). (Fig. 1B).

10.1128/mbio.03442-21.1 FIG S1 Immune response to the Sputnik V vaccine of seropositive participants at baseline. (A) IgG anti-SARS-CoV-2 spike levels quantified according to the WHO International Standard (*N *=* *30). Antibodies were measured at 21, 42, and 120 days after the first dose of vaccine (after dose 1, 21 days; after dose 2, 42 days). (B) Neutralizing titers measured at 50% inhibition for the pseudotyped virus (CoV2pp GFP). Measurements were made 21, 42, and 120 days after the first dose. Data were analyzed using a Wilcoxon matched-pair test. Statistical significance is shown with the following notations: **, *P < *0.01; NS, not significant. Download FIG S1, EPS file, 0.7 MB. Copyright © 2022 Gonzalez Lopez Ledesma et al. 2022 Gonzalez Lopez Ledesma et al. https://creativecommons.org/licenses/by/4.0/ This content is distributed under the terms of the Creative Commons Attribution 4.0 International license.

The data show that although the total amount of IgG anti-spike decreases more than 10-fold over a period of 6 months after Sputnik V vaccination, the neutralizing capacity in naive individuals showed only a 2-fold reduction. Analysis of the neutralization index calculated by dividing the neutralizing titer of each sample by its respective IgG anti-spike concentration (IU/ml) shows a significant index increase as a function of time (Fig. 1C), suggesting a process of antibody maturation during this period.

### Reduced VOC escape to neutralization over time after Sputnik V vaccination.

**FIG 2:**
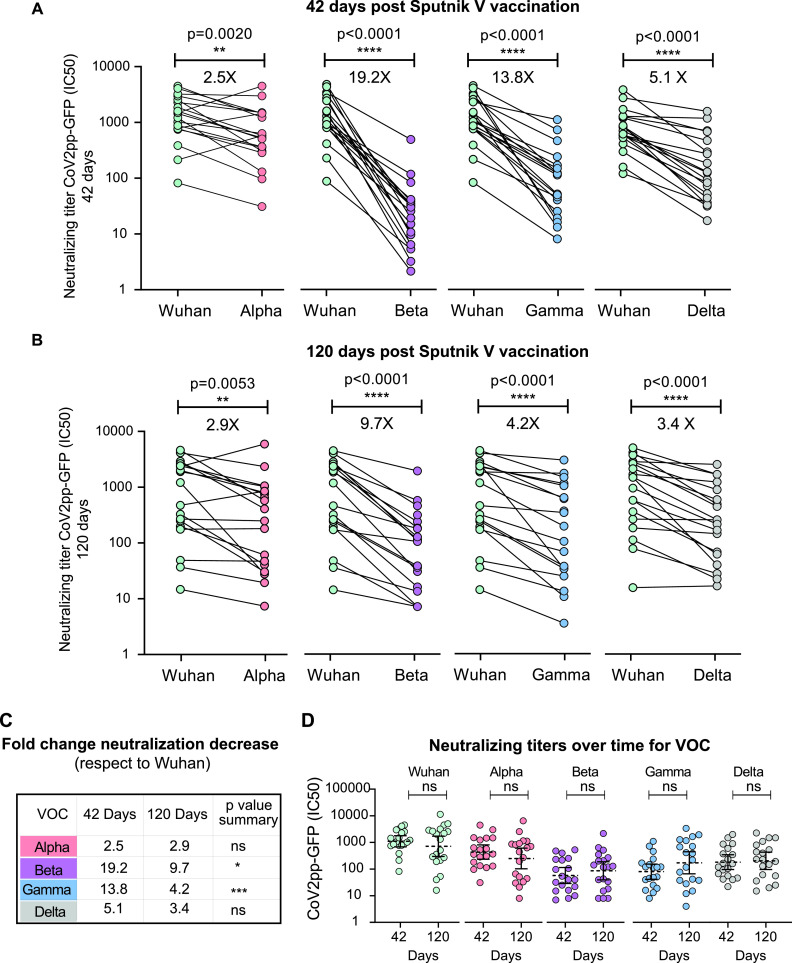
Longitudinal analysis of neutralizing capacities of serum samples from Sputnik-vaccinated participants for each variant of concern (VOC). Half-maximal neutralizing titers (IC_50_) against VOC using pseudotyped viruses (CoV2pp GFP), shown as fold change reduction normalized to the IC_50_ titer against the original virus (*N* = 19). Sera collected 42 (A) or 120 (B) days after the initial vaccination were used for neutralization assays with the Alpha, Beta, Gamma, and Delta variants, as indicated in each case. A Wilcoxon matched-pair test was used to analyze the data shown in panels A and B. (C) Comparison of fold change neutralization titer decreased for each VOC with respect to the Wuhan virus at 42 and 120 days after vaccination. The Mann-Whitney U test was used. The significance of the reduction is indicated on the right. (D) Neutralizing capacity at 42 and 120 days after initial vaccination for each variant is indicated. For nonpaired samples analysis in panel D the Mann-Whitney U test was used. Statistical significance is shown with the following notations: ****, *P < *0.0001; ***, *P < *0.001; *, *P < *0.05; ns, not significant.

We then evaluated the serum-neutralizing activity for circulating VOC elicited by Sputnik V vaccination. Viral infection inhibition was assessed using two systems, a VSV-based pseudotyped virus and the isolated SARS-CoV-2. The VSV-based system encoded GFP and was pseudotyped with the spike protein corresponding to the original SARS-CoV-2 (Wuhan) and the Alpha (lineage B.1.1.7), Beta (lineage B.1.351), Gamma (lineage P.1), and Delta (lineage B.1.617.2) variants, which were initially identified in the United Kingdom, South Africa, Manaos, and India, respectively. Serum samples collected 42 days after vaccination showed a 2.5- and 5.1-fold decrease in neutralizing activity against the Alpha and Delta variants compared with the Wuhan-related pseudotyped virus (*P = *0.002 and *P < *0,0001, respectively) (Fig. 2A). The samples were less effective at neutralizing the Beta and Gamma variants (19.2- and 13.8-fold reduction in neutralizing activity, *P < *0.0001 and *P < *0.0001, respectively).

Serum samples collected 120 days after vaccination showed a 2.9-, 9.7-, 4.2-, and 3.4-fold decrease in neutralizing activity against the Alpha, Beta, Gamma, and Delta variants, respectively, compared with the Wuhan virus (Fig. 2B). Interestingly, a significant increase in the relative neutralization capacity was observed over time (from 42 to 120 days after vaccination) for the Beta and Gamma VOC with respect to the Wuhan-related virus (Fig. 2C). In addition, the level of neutralizing capacities over time was maintained for all the variants tested (Fig. 2D).

**FIG 3:**
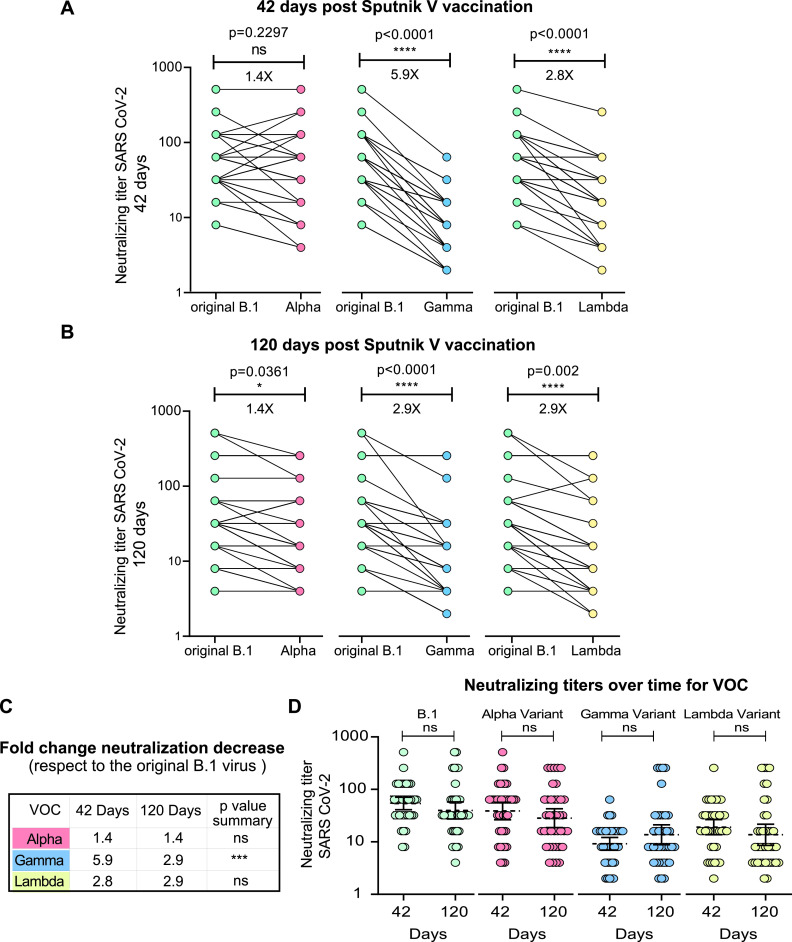
Longitudinal analysis of the neutralizing capacities of sera from Sputnik V-vaccinated individuals for each variant of concern (VOC) using viral isolates circulating in Argentina. The neutralizing titer (IC_90_) for VOC using a replicating SARS-CoV-2 was assessed and shown as fold change reduction with respect to that for the original B.1 virus. Neutralizing titers were defined as the highest serum dilution without any cytopathic effect on the monolayer. Sera (*N *=* *40) collected 42 days (A) or 120 days (B) after initial immunization were used for neutralization assays with Alpha, Gamma, and Lambda variants, as indicated in each case. Each sample in panels A and B is indicated with a dot, and the multiple lines from one dot correspond to different samples with the same neutralization titer. Wilcoxon matched-pair test was used in panels A and B. (C) Comparison of fold change neutralization titer decrease for each VOC with respect to the original B.1 virus at 42 and 120 days after vaccination. The Mann-Whitney U test was used. The significance of the reduction is indicated on the right. (D) Neutralizing capacity calculated at 42 and 120 days after initial immunization is shown for each variant. For nonpaired samples analysis in panel D the Mann-Whitney U test was used. Statistical significance is shown with the following notations: ****, *P < *0.0001; ***, *P < *0.001; *, *P < *0.05; ns, not significant.

The Lambda variant was initially detected in late December 2020 in South America (Andina, lineage C.37) (16). This novel sublineage within B.1.1.1, with a convergent deletion in the ORF1a gene (Δ3675–3677) and a novel deletion in the spike gene (Δ246–252, G75V, T76I, L452Q, F490S, T859N), rapidly spread in the region, replacing the Alpha variant and reaching frequencies in Argentina as high as 48% (17). The Lambda, Alpha, and Gamma variants are the main SARS-CoV-2 viruses currently circulating in Argentina. The antibody-neutralizing activity elicited by the Sputnik V vaccine was analyzed using the original local isolate B.1 (Wuhan related) virus and local isolates of the viral variants. A subset of 40 randomly selected group 1 volunteers was used for this analysis. Neutralizing titers were defined as the highest serum dilution that failed to elicit a cytopathic effect (CPE) on the cell monolayer (Fig. 3). Each sample is indicated with a dot, and the multiple lines in one dot correspond to different samples with the same neutralization titer.

The virus-neutralizing capacity of serum from Sputnik V-vaccinated individuals was slightly lower for the Lambda variant than for the original B.1 virus (2.8- and 2.9-fold change at 42 and 120 days after the first vaccination dose, respectively). For the Alpha variant, no significant (at 42 days after the first dose) or mild (at 120 days after the first dose) escape to neutralization was observed (Fig. 3A and B). In contrast, sera from vaccinated volunteers were less effective against the Gamma variant, showing a 5.9- and 2.9-fold reduction of inhibition in samples collected at 42 and 120 days after the initial vaccination, respectively (Fig. 3C). Neutralization capacity was maintained over time after Sputnik V vaccination for all SARS-CoV-2 variants analyzed (Fig. 3D). Together, these data suggest an improved cross-neutralization capacity, limiting VOC antibody escape to neutralization over time after vaccination.

## DISCUSSION

Unraveling the long-term kinetics of antibodies to SARS-CoV-2 and VOC in vaccinated individuals is important for understanding protective immunity against COVID-19 and for devising effective control measures. Here, we investigated the longitudinal humoral response in 118 volunteers vaccinated with Sputnik V. Our data indicate that anti-spike IgG antibodies wane over 6 months after vaccination with a minor loss of serum-neutralizing capacity against the original SARS-CoV-2 variant. The neutralizing capacity of the antibodies elicited by the vaccine was slightly or not significantly reduced for the Alpha, Lambda, and Delta variants, while partial escape to neutralization was observed for the Beta and Gamma variants. Antibodies elicited in individuals vaccinated with the Sputnik V vaccine exhibited increased cross-neutralization capacity over time to VOC. This might reflect the maturation of the antibody response induced by Sputnik V vaccination. Similar cross-neutralization increase for the original and VOC viruses was recently observed in longitudinal studies of individuals previously infected with SARS-CoV-2 (8) and of those harboring cloned neutralizing antibodies derived from convalescent donors (7, 9). These studies suggest that declining antibody titers are not indicative of declining protection. In summary, our studies support an increase of cross-neutralization to circulating SARS-CoV-2 variants, reducing the viral escape to neutralization, in the months following Sputnik V vaccination. Further studies to evaluate efficacy over time for VOC in vaccinated individuals will be necessary to define correlates of protection with antibody and neutralization titers.

## MATERIALS AND METHODS

### Patient and sample origin.

This study monitors the humoral immune response over time postimmunization with Sputnik V vaccine in 118 health care workers from Buenos Aires province, Argentina. Patient information is given in Table S1 in the supplemental material. Blood was collected by venipuncture into SST tubes (BD Sciences) for serum and stored at 20ºC.

### Ethics.

Study enrollment started in January 2021 and is ongoing. Ethical approval was obtained from the central committee of the Ministry of Health of Buenos Aires, and all participants provided written informed consent prior to collection of data and specimens (Cod#2021-00983502). All specimens were deidentified prior to processing and antibody testing for all serum specimens.

### Cohort description.

The cohort was 31.4% male and 68.6% female, with an average age of 49.3 years (range, 22 to 76 years). Information about ethnicity was not collected.

Sequential serum samples were collected at five time points, before vaccination (baseline) and at 21, 42, 120, and 180 days after the initial vaccination starting in January 2021. Volunteers were divided into two groups: without (group 1) or with (group 2) previous SARS-CoV-2 infection. Volunteers were classified according to the result of an enzyme-linked immunosorbent assay (ELISA) IgG anti-spike test at baseline.

### Cell lines.

Vero-CCL81 cells (ATCC) and 293T ACE2/TMPRSS2 cells (kindly provided by Benhur Lee) were used. Cells were cultured at 37°C in 5% CO_2_ in Dulbecco’s modified Eagle’s high-glucose medium (Thermo Fisher Scientific) supplemented with 10% fetal bovine serum (FBS) (GIBCO).

### Viruses.

SARS-CoV-2 pseudotyped particles (CoV2pp-GFP) expressing spike protein were generated in the Sean Whelan laboratory. The S gene of SARS-CoV-2 isolate Wuhan-Hu-1 (GenBank accession no. MN908947.3) and pseudotyped VSVs expressing variants of the SARS-CoV-2 spike, including B.1.1.7 (GenBank accession no. OU117158.1), B.1.351 (GenBank accession no. MZ212516.1), and P.1 (GISAID EPI_ISL_804823), were generated (15).

The B.1, Alpha (B.1.1.7), Gamma (P.1), and Lambda (C.37) isolates were obtained from nasopharyngeal isolates. All viruses were passaged only once in Vero cells. To confirm the substitutions of the respective variant and to evaluate stability during the passage, the whole genome was subjected to deep sequencing using an Illumina platform. All virus experiments were performed in approved biosafety level 3 facilities.

### SARS-CoV-2 pseudotyped VSV.

Briefly, pseudotyped VSVs were rescued by infecting BSRT7/5 cells with vaccinia virus vTF7-3 and subsequently transfecting them with T7-driven support plasmids encoding VSV N, P, L, G, and VSV genomic cDNAs. Supernatants were harvested 72 h postinfection, cellular debris was removed by centrifugation (5 min and 1,000 × *g*), and supernatants were passed through 0.22-μm filters. Supernatants were plaque purified on Vero-CCL81 cells. Individual clones were grown on Vero-CCL81 cells to generate P1 stocks. Working stocks were generated on Vero-CCL81 cells at 34°C. Viral stocks (VSV-eGFP-SARS-CoV-2), generated in the Sean Whelan laboratory, were amplified in our laboratory using 293T ACE2/TMPRSS2 cells at an MOI of 0.01 in Dulbecco’s modified Eagle’s medium containing 2% FBS at 37°C. Viral supernatants were harvested upon extensive CPE and GFP-positive cells. The medium was clarified by centrifugation at 1,000 × *g* for 5 min. Viral stocks were titrated by fluorescence-forming units per milliliter (FFU/ml) in the Vero cell line. Aliquots were maintained at −80°C.

### SARS-CoV-2.

SARS-CoV-2 ancestral reference strain 2019 (GISAID accession ID EPI_ISL_499083) B.1 was obtained from Sandra Gallegos (InViV working group). Alpha (GISAID accession ID EPI_ISL_2756558) and Gamma (GISAID accession ID EPI_ISL_2756556) variants were isolated at INBIRS from nasopharyngeal swabs. Lambda (hCoV-19/Argentina/PAIS-A0612/2021; GISAID accession ID EPI_ISL_3320903) variant was isolated at INBIRS from a sample of nasopharyngeal swab kindly transferred by M. Viegas and P. Pais. Virus was amplified in Vero E6 cells, and viral stock identity was confirmed by whole-genome sequencing in an Illumina sequencer. Nucleic acid sequence for each viral stock was uploaded to GISAID and completely matched reference sequences for each variant, discarding acquisition of mutations during isolation and amplification processes. Work with SARS-CoV-2 was approved by the INBIRS Institutional Biosafety Committee at biosafety level 3 with negative pressure.

### Sequencing of the S gene.

Viral RNA was extracted from VSV-SARS-CoV-2 mutant viruses using TRIzol LS reagent (Thermo Fisher Scientific), and S was amplified using M-MLV reverse transcriptase (Thermo Fisher Scientific). The mutations were identified by Sanger sequencing (Applied Biosystems).

### SARS-CoV-2 antibody ELISA.

Antibodies to SARS-CoV-2 spike protein were detected using an established commercially available two-step ELISA (COVIDAR). We have previously described the development of the ELISA (13). Briefly, the assay uses plates coated with a mixture of spike and the receptor binding domain (RBD). The viral proteins were purified from transfected FreeStyle 293-F suspension cells using HisTrap excel columns. The conjugated monoclonal antibody used for human IgG detection in the COVIDAR ELISA is G18-145, which specifically binds to the heavy chain of all four human immunoglobulin G subclasses: IgG1, IgG2, IgG3, and IgG4.

The IgG concentration of each sample, expressed in international units per milliliter (14), was calculated by extrapolation of the optical density at 450 nm (OD_450_) on a calibration curve. For construction of the calibration curve, we determined the OD_450_ of serial dilutions of the WHO International Standard for anti-SARS-CoV-2 immunoglobulin. The linear range used was an OD_450_ of 0.2 to 1.5. Therefore, we performed serial dilutions of the samples to find conditions where the OD_450_ of each sample fit adequately in the linear range.

### SARS-CoV-2 spike pseudotyped VSV neutralization assay.

To compare the neutralizing activity of volunteer’s sera against coronaviruses, neutralization assays were carried out with SARS-CoV-2 pseudotyped particles (CoV2pp-GFP), generated in the Sean Whelan laboratory (15). CoV2pp-GFP carries vesicular stomatitis virus as the viral backbone, and the glycoprotein gene (G) was replaced with the full-length wild-type or VOC spike protein of SARS-CoV-2 (VSV-eGFP-SARS-CoV-2). Vero cells were used for these assays. Cells were maintained with Dulbecco’s modified Eagle’s medium (DMEM) high glucose with 10% FBS and were seeded in a 96-well plate the day before infection. Patient sera were heat inactivated at 56°C for 30 min and serially diluted in DMEM high-glucose medium. Serum neutralizations were performed by first diluting the inactivated sample 2-fold and continuing with a 2-fold serial dilution. A pretitrated amount of pseudotyped particles was incubated with a 2-fold serial dilution of patient sera for 1 h at 37°C prior to infection. Subsequently, cells were fixed in 4% formaldehyde containing 2 mg/ml 4′,6-diamidino-2-phenylindole (DAPI) nuclear stain (Invitrogen) for 1 h at room temperature, and fixative was replaced with PBS. Images were acquired with the InCell 2000 Analyzer (GE Healthcare) automated microscope in both the DAPI and fluorescein isothiocyanate (FITC) channels to visualize nuclei and infected cells (i.e., eGFP-positive cells), respectively (4× objective, 4 fields per well, covering the entire well). Images were analyzed using the multitarget analysis module of the InCell Analyzer 2000 workstation software (GE Healthcare). GFP-positive cells were identified in the FITC channel using the top-hat segmentation method and subsequently counted within the InCell Workstation software. Absolute inhibitory concentration (absIC) values were calculated for all patient serum samples by modeling a 4-parameter logistic (4PL) regression with GraphPad Prism 8. The 4PL model describes the sigmoid-shaped response pattern. For clarity, it is assumed that the response can be expressed so that the slope increases as the concentration increase. absIC was calculated as the corresponding point between the 0% and 100% assay controls. Fifty percent inhibition was defined by the controls for all samples on the same plate. For example, the absIC50 would be the point at which the curve matches inhibition equal to exactly 50% of the 100% assay control relative to the assay minimum. A 4PL regression with GraphPad Prism 8 was used.

### SARS-CoV-2 neutralization assay.

Serum samples were heat inactivated at 56°C for 30 min and serial dilutions from 1/2 to 1/8,192 were incubated for 1 h at 37°C in the presence of ancestral or variants of SARS-CoV-2 in DMEM, 2% FBS. Fifty microliters of the mixture was then deposited over Vero cell monolayers for an hour at 37°C (MOI, 0.01). Infectious medium was removed and replaced for DMEM, 2% FBS. After 72 h, cells were fixed with 4% paraformaldehyde (4°C, 20 min) and stained with crystal violet solution in methanol. The CPE of the virus on the cell monolayer was assessed visually. If even minor damage to the monolayer was observed in the well, the well was considered a well with a manifestation of CPE. Neutralization titer was defined as the highest serum dilution without any CPE in two of three replicable wells.

### Quantification and statistical analysis.

Antibody concentration, neutralizing titer, and neutralizing potency index from volunteers of the same group were analyzed collectively. Neutralization assays were performed in biological duplicates.

All statistical tests and plots were performed using GraphPad Prism 8.0 software. Comparison on nonpaired determinations of antibody concentration and neutralizing titer were made using two-tailed Mann-Whitney U test in Fig. 2C and D and 3C and D. Comparisons of antibody concentration and neutralizing titer were made using two-tailed Wilcoxon matched-pair test in Fig. 1A and B, 2A and B, and 3A and B and Fig. S1. Statistical significance is shown in the figure legends with the following notations: ****, *P* < 0.0001; ***, *P* < 0.001; **, *P* < 0.01; *, *P* < 0.05; ns, not significant. Geometric means with 95% confidence intervals were calculated for Fig. 1A and B, 2A and B, and 3A and B and Fig. S1.

### Data availability.

The data sets generated and/or analyzed during the current study are available in the Mendeley Data repository at https://doi.org/10.17632/v2ksr58dcv.1.

## References

[1] World Health Organization. 2021. WHO coronavirus (COVID-19) dashboard with vaccination data. World Health Organization, Geneva, Switzerland.

[2] Khoury DS, Cromer D, Reynaldi A, Schlub TE, Wheatley AK, Juno JA, Subbarao K, Kent SJ, Triccas JA, Davenport MP. 2021. Neutralizing antibody levels are highly predictive of immune protection from symptomatic SARS-CoV-2 infection. Nat Med 27:1205–1207. doi:10.1038/s41591-021-01377-8. 34002089

[3] Krammer F. 2021. A correlate of protection for SARS-CoV-2 vaccines is urgently needed. Nat Med 27:1147–1148. doi:10.1038/s41591-021-01432-4. 34239135

[4] Earle KA, Ambrosino DM, Fiore-Gartland A, Goldblatt D, Gilbert PB, Siber GR, Dull P, Plotkin SA. 2021. Evidence for antibody as a protective correlate for COVID-19 vaccines. Vaccine 39:4423–4428. doi:10.1016/j.vaccine.2021.05.063. 34210573 PMC8142841

[5] Wang Z, Muecksch F, Schaefer-Babajew D, Finkin S, Viant C, Gaebler C, Hoffmann H-H, Barnes CO, Cipolla M, Ramos V, Oliveira TY, Cho A, Schmidt F, Da Silva J, Bednarski E, Aguado L, Yee J, Daga M, Turroja M, Millard KG, Jankovic M, Gazumyan A, Zhao Z, Rice CM, Bieniasz PD, Caskey M, Hatziioannou T, Nussenzweig MC. 2021. Naturally enhanced neutralizing breadth against SARS-CoV-2 one year after infection. Nature 595:426–431. doi:10.1038/s41586-021-03696-9. 34126625 PMC8277577

[6] Dispinseri S, Secchi M, Pirillo MF, Tolazzi M, Borghi M, Brigatti C, De Angelis ML, Baratella M, Bazzigaluppi E, Venturi G, Sironi F, Canitano A, Marzinotto I, Tresoldi C, Ciceri F, Piemonti L, Negri D, Cara A, Lampasona V, Scarlatti G. 2021. Neutralizing antibody responses to SARS-CoV-2 in symptomatic COVID-19 is persistent and critical for survival. Nat Commun 12:2670. doi:10.1038/s41467-021-22958-8. 33976165 PMC8113594

[7] Gaebler C, Wang Z, Lorenzi JCC, Muecksch F, Finkin S, Tokuyama M, Cho A, Jankovic M, Schaefer-Babajew D, Oliveira TY, Cipolla M, Viant C, Barnes CO, Bram Y, Breton G, Hägglöf T, Mendoza P, Hurley A, Turroja M, Gordon K, Millard KG, Ramos V, Schmidt F, Weisblum Y, Jha D, Tankelevich M, Martinez-Delgado G, Yee J, Patel R, Dizon J, Unson-O'Brien C, Shimeliovich I, Robbiani DF, Zhao Z, Gazumyan A, Schwartz RE, Hatziioannou T, Bjorkman PJ, Mehandru S, Bieniasz PD, Caskey M, Nussenzweig MC. 2021. Evolution of antibody immunity to SARS-CoV-2. Nature 591:639–644. doi:10.1038/s41586-021-03207-w. 33461210 PMC8221082

[8] Moriyama S, Adachi Y, Sato T, Tonouchi K, Sun L, Fukushi S, Yamada S, Kinoshita H, Nojima K, Kanno T, Tobiume M, Ishijima K, Kuroda Y, Park E-S, Onodera T, Matsumura T, Takano T, Terahara K, Isogawa M, Nishiyama A, Kawana-Tachikawa A, Shinkai M, Tachikawa N, Nakamura S, Okai T, Okuma K, Matano T, Fujimoto T, Maeda K, Ohnishi M, Wakita T, Suzuki T, Takahashi Y. 2021. Temporal maturation of neutralizing antibodies in COVID-19 convalescent individuals improves potency and breadth to circulating SARS-CoV-2 variants. Immunity 54:1841–1852.e4. doi:10.1016/j.immuni.2021.06.015. 34246326 PMC8249673

[9] Muecksch F, Weisblum Y, Barnes CO, Schmidt F, Schaefer-Babajew D, Wang Z, Lorenzi JCC, Flyak AI, DeLaitsch AT, Huey-Tubman KE, Hou S, Schiffer CA, Gaebler C, Da Silva J, Poston D, Finkin S, Cho A, Cipolla M, Oliveira TY, Millard KG, Ramos V, Gazumyan A, Rutkowska M, Caskey M, Nussenzweig MC, Bjorkman PJ, Hatziioannou T, Bieniasz PD. 2021. Affinity maturation of SARS-CoV-2 neutralizing antibodies confers potency, breadth, and resilience to viral escape mutations. Immunity 54:1853–1816. doi:10.1016/j.immuni.2021.07.008. 34331873 PMC8323339

[10] Logunov DY, Dolzhikova IV, Zubkova OV, Tukhvatullin AI, Shcheblyakov DV, Dzharullaeva AS, Grousova DM, Erokhova AS, Kovyrshina AV, Botikov AG, Izhaeva FM, Popova O, Ozharovskaya TA, Esmagambetov IB, Favorskaya IA, Zrelkin DI, Voronina DV, Shcherbinin DN, Semikhin AS, Simakova YV, Tokarskaya EA, Lubenets NL, Egorova DA, Shmarov MM, Nikitenko NA, Morozova LF, Smolyarchuk EA, Kryukov EV, Babira VF, Borisevich SV, Naroditsky BS, Gintsburg AL. 2020. Safety and immunogenicity of an rAd26 and rAd5 vector-based heterologous prime-boost COVID-19 vaccine in two formulations: two open, non-randomised phase 1/2 studies from Russia. Lancet 396:887–897. doi:10.1016/S0140-6736(20)31866-3. 32896291 PMC7471804

[11] Logunov DY, Dolzhikova IV, Shcheblyakov DV, Tukhvatulin AI, Zubkova OV, Dzharullaeva AS, Kovyrshina AV, Lubenets NL, Grousova DM, Erokhova AS, Botikov AG, Izhaeva FM, Popova O, Ozharovskaya TA, Esmagambetov IB, Favorskaya IA, Zrelkin DI, Voronina DV, Shcherbinin DN, Semikhin AS, Simakova YV, Tokarskaya EA, Egorova DA, Shmarov MM, Nikitenko NA, Gushchin VA, Smolyarchuk EA, Zyryanov SK, Borisevich SV, Naroditsky BS, Gintsburg AL. 2021. Safety and efficacy of an rAd26 and rAd5 vector-based heterologous prime-boost COVID-19 vaccine: an interim analysis of a randomised controlled phase 3 trial in Russia. Lancet 397:671–681. doi:10.1016/S0140-6736(21)00234-8. 33545094 PMC7852454

[12] Rossi AH, Ojeda DS, Varese A, Sanchez L, Gonzalez Lopez Ledesma MM, Mazzitelli I, Juliá AA, Rouco SO, Pallarés HM, Costa Navarro GS, Rasetto N, Garcia CI, Wenker SD, Ramis LY, Bialer MG, Jose de Leone M, Hernando CE, Sosa S, Bianchimano L, Rios A, Treffinger Cienfuegos MS, Caramelo JJ, Longueira Y, Laufer N, Alvarez D, Carradori J, Pedrozza D, Rima A, Echegoyen C, Ercole R, Gelpi P, Marchetti S, Zubieta M, Docena G, Kreplak N, Yanovsky M, Geffner J, Pifano M, Gamarnik AV. 2021. Sputnik V vaccine elicits seroconversion and neutralizing capacity to SARS CoV-2 after a single dose. Cell Rep Med 2:100359. doi:10.1016/j.xcrm.2021.100359. 34308389 PMC8266543

[13] Ojeda DS, Gonzalez Lopez Ledesma MM, Pallarés HM, Costa Navarro GS, Sanchez L, Perazzi B, Villordo SM, Alvarez DE, Echavarria M, Oguntuyo KY, Stevens CS, Lee B, Carradori J, Caramelo JJ, Yanovsky MJ, Gamarnik AV, BioBanco Working Group. 2021. Emergency response for evaluating SARS-CoV-2 immune status, seroprevalence and convalescent plasma in Argentina. PLoS Pathog 17:e1009161. doi:10.1371/journal.ppat.1009161. 33444413 PMC7808630

[14] Kristiansen PA, Page M, Bernasconi V, Mattiuzzo G, Dull P, Makar K, Plotkin S, Knezevic I. 2021. WHO international standard for anti-SARS-CoV-2 immunoglobulin. Lancet 397:1347–1348. doi:10.1016/S0140-6736(21)00527-4. 33770519 PMC7987302

[15] Case JB, Rothlauf PW, Chen RE, Liu Z, Zhao H, Kim AS, Bloyet LM, Zeng Q, Tahan S, Droit L, Ilagan MXG, Tartell MA, Amarasinghe G, Henderson JP, Miersch S, Ustav M, Sidhu S, Virgin HW, Wang D, Ding S, Corti D, Theel ES, Fremont DH, Diamond MS, Whelan SPJ. 2002. Neutralizing antibody and soluble ACE2 inhibition of a replication-competent VSV-SARS-CoV-2 and a clinical isolate of SARS-CoV-2. Cell Host Microbe 28:475–485. doi:10.1016/j.chom.2020.06.021. PMC7332453 32735849

[16] Darvishi M, Rahimi F, Talebi Bezmin Abadi A. 2021. SARS-CoV-2 Lambda (C.37): an emerging variant of concern? Gene Rep 25:101378. doi:10.1016/j.genrep.2021.101378. 34632160 PMC8487850

[17] País P. 2021. Reporte no 15: vigilancia activa de variantes de SARS-CoV-2 en la CABA, provincias de Buenos Aires, Chaco, Córdoba, La Pampa, Neuquén y Santa Fe. Actualización 2020:1–11.

