# Supplementary material for: Longitudinal Study after Sputnik V Vaccination Shows Durable SARS-CoV-2 Neutralizing Antibodies and Reduced Viral Variant Escape to Neutralization over Time
Source: mBio. 2022 Jan 25;13(1):e03442-21. doi: 10.1128/mbio.03442-21 (PMC8787469; doi:10.1128/mbio.03442-21)
Supplement: TABLE S1 [file mbio.03442-21-st001.docx]

Supplementary Table 1: Patient information. Age, gender and group classification.

| **ID** | **Sex** | **Age** | **Group clasification** |
| --- | --- | --- | --- |
| 1 | F | 76 | 1 |
| 2 | M | 74 | 1 |
| 3 | M | 74 | 1 |
| 4 | M | 68 | 1 |
| 5 | M | 67 | 1 |
| 6 | F | 67 | 1 |
| 7 | M | 64 | 1 |
| 8 | M | 65 | 1 |
| 9 | M | 66 | 1 |
| 10 | F | 63 | 1 |
| 11 | F | 63 | 1 |
| 12 | F | 62 | 1 |
| 13 | M | 65 | 1 |
| 14 | M | 63 | 1 |
| 15 | F | 62 | 1 |
| 16 | M | 62 | 1 |
| 17 | F | 62 | 1 |
| 18 | M | 62 | 1 |
| 19 | F | 62 | 1 |
| 20 | F | 62 | 1 |
| 21 | F | 62 | 1 |
| 22 | M | 61 | 1 |
| 23 | M | 62 | 1 |
| 24 | F | 60 | 1 |
| 25 | F | 61 | 1 |
| 26 | M | 60 | 1 |
| 27 | F | 60 | 1 |
| 28 | F | 59 | 1 |
| 29 | F | 59 | 1 |
| 30 | F | 58 | 1 |
| 31 | M | 56 | 1 |
| 32 | F | 57 | 1 |
| 33 | F | 55 | 1 |
| 34 | F | 55 | 1 |
| 35 | M | 55 | 1 |
| 36 | M | 54 | 1 |
| 37 | M | 54 | 1 |
| 38 | M | 53 | 1 |
| 39 | F | 50 | 1 |
| 40 | F | 52 | 1 |
| 41 | M | 52 | 1 |
| 42 | F | 52 | 1 |
| 43 | F | 52 | 1 |
| 44 | F | 52 | 1 |
| 45 | M | 52 | 1 |
| 46 | M | 49 | 1 |
| 47 | F | 49 | 1 |
| 48 | F | 49 | 1 |
| 49 | F | 48 | 1 |
| 50 | F | 48 | 1 |
| 51 | M | 47 | 1 |
| 52 | F | 46 | 1 |
| 53 | F | 47 | 1 |
| 54 | F | 47 | 1 |
| 55 | F | 46 | 1 |
| 56 | F | 45 | 1 |
| 57 | F | 45 | 1 |
| 58 | F | 44 | 1 |
| 59 | M | 42 | 1 |
| 60 | F | 42 | 1 |
| 61 | F | 42 | 1 |
| 62 | F | 42 | 1 |
| 63 | F | 42 | 1 |
| 64 | F | 42 | 1 |
| 65 | M | 41 | 1 |
| 66 | F | 39 | 1 |
| 67 | F | 39 | 1 |
| 68 | M | 39 | 1 |
| 69 | F | 38 | 1 |
| 70 | M | 38 | 1 |
| 71 | F | 38 | 1 |
| 72 | F | 36 | 1 |
| 73 | M | 36 | 1 |
| 74 | F | 36 | 1 |
| 75 | F | 35 | 1 |
| 76 | M | 34 | 1 |
| 77 | M | 35 | 1 |
| 78 | F | 34 | 1 |
| 79 | F | 34 | 1 |
| 80 | F | 33 | 1 |
| 81 | F | 32 | 1 |
| 82 | F | 27 | 1 |
| 83 | F | 28 | 1 |
| 84 | F | 29 | 1 |
| 85 | F | 27 | 1 |
| 86 | F | 26 | 1 |
| 87 | F | 26 | 1 |
| 88 | F | 22 | 1 |
| 89 | M | 60 | 2 |
| 90 | M | 26 | 2 |
| 91 | F | 43 | 2 |
| 92 | F | 64 | 2 |
| 93 | F | 42 | 2 |
| 94 | M | 60 | 2 |
| 95 | F | 36 | 2 |
| 96 | M | 65 | 2 |
| 97 | M | 24 | 2 |
| 98 | F | 49 | 2 |
| 99 | F | 28 | 2 |
| 100 | F | 50 | 2 |
| 101 | F | 48 | 2 |
| 102 | M | 50 | 2 |
| 103 | F | 55 | 2 |
| 104 | F | 27 | 2 |
| 105 | F | 58 | 2 |
| 106 | F | 45 | 2 |
| 107 | F | 47 | 2 |
| 108 | F | 36 | 2 |
| 109 | F | 46 | 2 |
| 110 | F | 39 | 2 |
| 111 | F | 65 | 2 |
| 112 | F | 52 | 2 |
| 113 | F | 60 | 2 |
| 114 | F | 68 | 2 |
| 115 | F | 30 | 2 |
| 116 | F | 60 | 2 |
| 117 | M | 57 | 2 |
| 118 | F | 51 | 2 |
